# Time-Course Study of the Transcriptome of Peripheral Blood Mononuclear Cells (PBMCs) from Sheep Infected with *Fasciola hepatica*

**DOI:** 10.1371/journal.pone.0159194

**Published:** 2016-07-20

**Authors:** Cristian A. Alvarez Rojas, Jean-Pierre Scheerlinck, Brendan R. E. Ansell, Ross S. Hall, Robin B. Gasser, Aaron R. Jex

**Affiliations:** 1 Centre for Animal Biotechnology, Faculty of Veterinary and Agricultural Sciences, The University of Melbourne, Parkville, Victoria, 3010, Australia; 2 Faculty of Veterinary and Agricultural Sciences, The University of Melbourne, Parkville, Victoria, 3010, Australia; 3 Population Health and Immunity, Walter and Eliza Hall Institute, Parkville, 3052, Australia; University of Minnesota, UNITED STATES

## Abstract

*Fasciola hepatica* is a parasitic trematode that infects a wide range of mammalian hosts, including livestock and humans, in temperate and tropical regions globally. This trematode causes the disease fascioliasis, which consists of an acute phase (≤ 12 weeks) during which juvenile parasites migrate through the host liver tissues, and a chronic phase (> 12 weeks) following the establishment of adult parasites in the liver bile ducts. Few studies have explored the progression of the host response over the course of *Fasciola* infection in the same animals. In this study, we characterized transcriptomic changes in peripheral blood mononuclear cells (PBMCs) collected from sheep at three time points over the first eight weeks of infection relative to uninfected controls. In total, 183 and 76 genes were found to be differentially transcribed at two and eight weeks post-infection respectively. Functional and pathway analysis of differentially transcribed genes revealed changes related to T-cell activation that may underpin a Th2-biased immune response against this parasite. This first insight into the dynamics of host responses during the early stages of infection improves the understanding of the pathogenesis of acute fascioliasis, informs vaccine development and presents a set of PBMC markers with diagnostic potential.

## Introduction

*Fasciola hepatica*, commonly known as liver fluke, is responsible for acute and chronic liver disease (= fascioliasis) in various mammals worldwide, including humans. The disease has important consequences in infected livestock due to losses arising from reduced productivity and fertility, mortalities, failure to thrive and the condemnation of livers [1]. The infection establishes via oral ingestion of metacercariae, usually encysted on aquatic vegetation. Upon passage through the digestive tract, the juvenile stage of the parasite emerges in the small intestine, penetrates the gut wall and proceeds to migrate to and through the liver of the host animal [2].

During host invasion, *F*. *hepatica* secretes a potent mix of digestive proteases, allowing it to undertake migration through and feed on host tissues, particularly liver parenchyma, before reaching its final destination, the bile ducts. This developmental process takes approximately six to 12 weeks [2]. At sufficient infection intensity, symptoms of acute fascioliasis appear, especially in sheep, as severe abdominal pain, blood-loss and associated anaemia/hypoproteinaemia. Even at low infection intensities, livers of infected sheep may show signs of inflammation, haemorrhage and tissue necrosis. Once established in the bile duct, *F*. *hepatica* can survive as adults for several years, leading to a chronic immunological response, severe cholangitis and fibrosis of the bile ducts. At sufficient intensity, such chronic infections are often associated with blockage of the bile ducts and concomitant distension of the gall bladder, as well as atrophy of liver parenchyma, resulting from tissues unable to recover from the initial juvenile assault [2, 3].

*Fasciola* infection is associated with a non-inflammatory Th2-biased response [4–6], partially regulated by “parasite-driven” immunomodulation [7–10]. Previous investigations have followed humoral and cellular responses to *Fasciola* infection in sheep and cattle using histopathological and immunological tools [7, 11–16]. Other investigations have focused on the detection of specific interleukins or other immune proteins (i.e. IFN-γ, IL-4, IL-10 and TGF-β1) from PBMCs in response to excretory/secretory (ES) products from the parasite in sheep [7, 8], cattle [10, 17] and mice [10]. Recently, Rojas-Caraballo et al. [18] followed the progression of the host response to *Fasciola* infection using RNA microarray analysis; however, though informative, this work was conducted in mice, an experimental host. On other hand, investigations on the natural host have provide evidence of the involvement of Galectins interacting with the parasite [19, 20]. Despite this research, our understanding of how the host response changes over time, particularly with respect to acute and chronic stages of infection, remains limited.

In a previous study, we examined the transcriptomic responses of liver tissue in sheep against those experimentally infected with *F*. *hepatica* for 8 weeks, and defined the involvement of specific genes associated with the host’s metabolism, immune response and tissue repair/regeneration, highlighting an apparent overlapping function of many genes involved in these processes [21]. In the present study, we explore transcriptomic changes in PBMCs taken from the same sheep before infection, and 2 and 8 weeks following experimental infection, and compare these changes to uninfected controls. We aimed to provide a detailed analysis of the transcription of genes involved in the complex immune networks in PBMCs and to gain a better understanding of the progression of the immune response of sheep to this parasite which might assist efforts to develop vaccines against *F*. *hepatica*.

## Material and Methods

### Animals and experimental design

All animal experimental procedures were approved by the Ethics Committee of the Faculty of Veterinary and Agricultural Sciences, The University of Melbourne (Ethics application ID 1312938.1). Four female Merino sheep (< 6months of age) received a single oral individual dose of 180 metacercariae of *F*. *hepatica* purchased from Baldwin Aquatics Inc., Oregon, USA. Four additional female animals of the same age and breeding stock were maintained as controls and kept in a separate raised pen in the same facility. All animals were verified helminth free by faecal inspection prior to the start of the study. Individual sheep were clinically monitored on a weekly basis checking for general condition and alert status. No signs of illness nor mortality was observed prior to the experimental endpoint. All food, water and animal management practices were consistent for infected and control animals throughout the study. Eight weeks after infection, each animal was euthanized by intravenous injection of pentobarbitone sodium (172.5 mg/kg) and immediately necropsied.

### PBMC isolation

Forty ml of blood were obtained weekly from the jugular vein of each animal (control and infected groups), starting from one day prior to the experimental infection. PBMCs were isolated before infection and 2 and 8 weeks post infection (WPI), as described previously [22]. Briefly, blood was collected in the presence of 100 μl of heparin (5000 units/ml) and centrifuged for 20 min at 600*g*, the buffy coat was collected and then diluted with 10 ml of phosphate-buffered saline (PBS) before being under-laid with 2 ml of Ficoll (1.077 specific gravity; Pharmacia Biotech, Sweden) and centrifuged for 30 min at 100*g*. The lymphocytes were collected and washed four times in PBS and stored in RNAlater® (Life Technologies, United States) following the manufacturer’s instructions.

### RNA extraction and library preparation

PBMCs from each animal were collected at the above mentioned time-points and centrifuged at 600*g* for 5 min. An aliquot of the pelleted cells (200 μl/sample) was washed once in PBS, and TriPure reagent (Roche Diagnostics, Switzerland) was added. RNA was isolated following the manufacturer’s instructions. Purified RNA was resuspended in RNAse-free water (Life technologies). RNA quality was assessed using a Bioanalyzer 2100 (Agilent, USA); samples with an RNA quality indicator (RQI) of ≥8 were used for sequencing. Library preparation and RNA sequencing were performed as described previously [21].

### Bioinformatic analysis

Paired-end RNA-seq reads for each sample/replicate were filtered for quality and adapter-trimmed using Trimmomatic software [23] (sliding window: 4 bp, leading and trailing: 3 bp, minimum read length: 100 bp; Phred quality: 25). Filtered reads were subjected to K-mer correction using the program corrector [24]. Data have been deposited in the Sequence Read Archive (SRA) from the National Center for Biotechnology Information (NCBI; SubmissionID: SUB1665337, BioProject ID: PRJNA327701). We used two strategies to define differentially transcribed genes (DTGs) in NOISeq. (1) First, because the immune response can vary significantly from individual to individual, we analyzed variability within the same animal two (T1) and eight weeks (T2) post infection, compared with the pre-infection timepoint (for control and infected animals) using NOISeq-sim (k = 0.5, q = 0.8). We filtered these DTGs and retained the genes that were differentially transcribed in at least 3 of 4 animals at each time point. Finally, we discarded any DTGs from infected animals found to be differentially transcribed over the same time-points in the uninfected controls. (2) In a second analysis, we treated infected and uninfected animals as biological replicates of the experimental and control groups, respectively. For this, we NOISeq-bio (k = 0.5, q = 0.9) comparing transcripts per million (TPM) values at 2 and 8 WPI in infected animals to the uninfected control animals. We kept for further analysis only those genes identified as being differentially transcribed at statistical significance using both analytical strategies.

### Gene Ontology and KEGG analysis

All gene functional enrichment analyses relating to DTGs defined for infected animals were assessed based on the gene models provided in the *Ovis aries* genome assembly version Oar_v3.1 (GCA_000298735.1) hosted by Ensembl [25], and gene names and descriptions as they were annotated in Biomart application [26] as of 01/04/2016. Over-representation of GO terms in up- and down-regulated DTGs at each time-point and with respect to the control animals were identified using PANTHER [27] (adjusted P<0.05). DTGs were assigned to conserved biological pathways in the Kyoto Encyclopedia of Genes and Genomes (KEGG) using Kobas [28], using an expected value of 10^−5^. Pathway-enrichment analysis was conducted using the Fisher hyper-geometric mean tests to identify significantly enriched KEGG pathways and enzyme hierarchies for DTGs relative to all KEGG-annotated genes [29, 30].

## Results and Discussion

Experimental infection was successful in all the four animals showing a similar response with an elevated number of eosinophils since the 4th week after infection (as previously shown in a previous study [21]. A detailed account of the RNA-seq data generated in this study can be found in **S1 Table**. In total more than 100 million reads were mapped to the *Ovis aries* genome; a mean of 58% of these reads mapped to recognized coding gene models. Because immune responses within individual animals are dependent in part on the genetics of the animal [31], we considered the possibility that it may not be appropriate to assess each animal as a biological replicate. We, therefore, adopted two strategies for our RNA-seq analyses; (Strategy1) in which we assessed each animal as an independent experiment using Noiseq-sim (which was developed for single replicate RNA-seq; see methods for details) and (Strategy 2) in which we treated infected and uninfected animals as biological replicates of an experimental *versus* control group using Noiseqbio.

**Fig 1:**
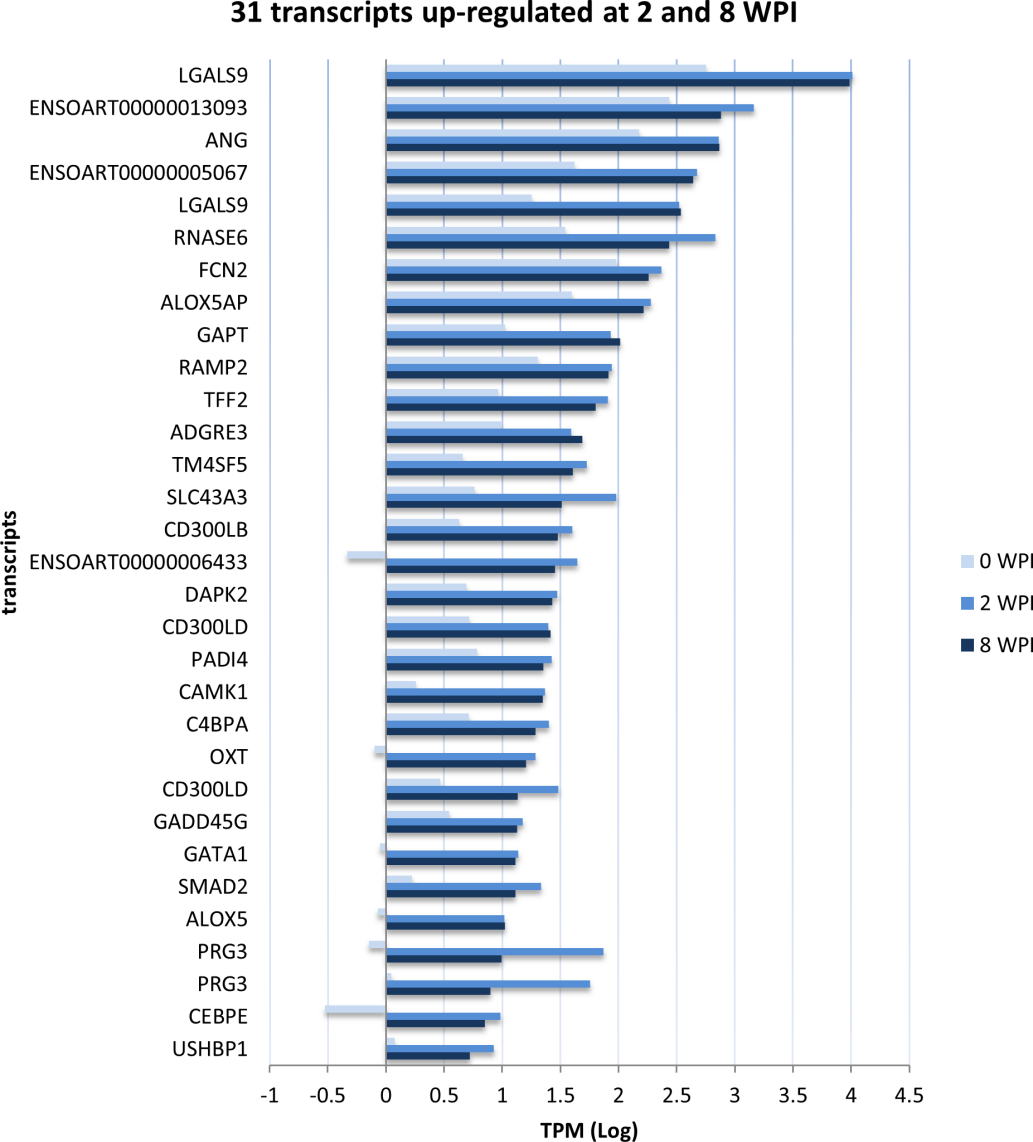
Genes that were found to be UP-regulated in ovine PBMCs at 2 and 8 weeks post infection with *Fasciola hepatica* compared with transcription at pre infection time (T0). Analysis was performed using the package NOISeq.

Based on this approach, with strategy 1, we identified 216 differentially transcribed genes (DTGs; 149 up- and 67 down-regulated) at 2 WPI and 95 DTGs (78 up- and 17 down-regulated) in at least 3 of the 4 infected animals and 0 of the 4 control animals at contemporary time-points (**S2 Table**). With strategy 2, we identified 4,819 (2,187 up- and 2,632 down-regulated) and 2,537 (1,627 up and 910 down-regulated) DTGs at 2 and 8 WPI, respectively (**S2 Table**). In filtering these results for DTGs identified using both strategies 1 and 2, we identified a consensus set of 183 DTGs (135 up- and 48 down-regulated; **S1 Fig**) and 76 DTGs (63 up- and 13 down-regulated; **S1 Fig**) at 2 and 8 WPI respectively (**Table 1**). Among the consensus set of DTGs (henceforth referred to only as ‘DTGs’) at 2 and 8 WPI, we found 31 up-regulated at both time-points (**Fig 1;** i.e., continuously up-regulated over the course of the infection), with a single gene down-regulated at both time points (transcript ENSOART00000001087); this gene encodes the exocyst complex component 6 (EXOC6) involved in the docking of exocytic vesicles with fusion sites on the plasma membrane. Detailed information on the genes shown to be differentially transcribed, with their respective TPM values, can be found in **S3 Table and S4 Table** for up- and down- regulated genes at 2 WPI respectively and **S5 Table and** **S6** Table for up- and down- regulated genes at 8 WPI, respectively.

**Table 1:** Final number of differentially transcribed genes (DTGs) in ovine peripheral blood mononuclear cells (PBMCs) at 2 and 8 Weeks post infection (WPI) after infection with *Fasciola hepatica*.

Condition	2 WPI	8 WPI
UP-regulated	135	63
DOWN-regulated	48	13
Total	183	76

**Fig 2:**
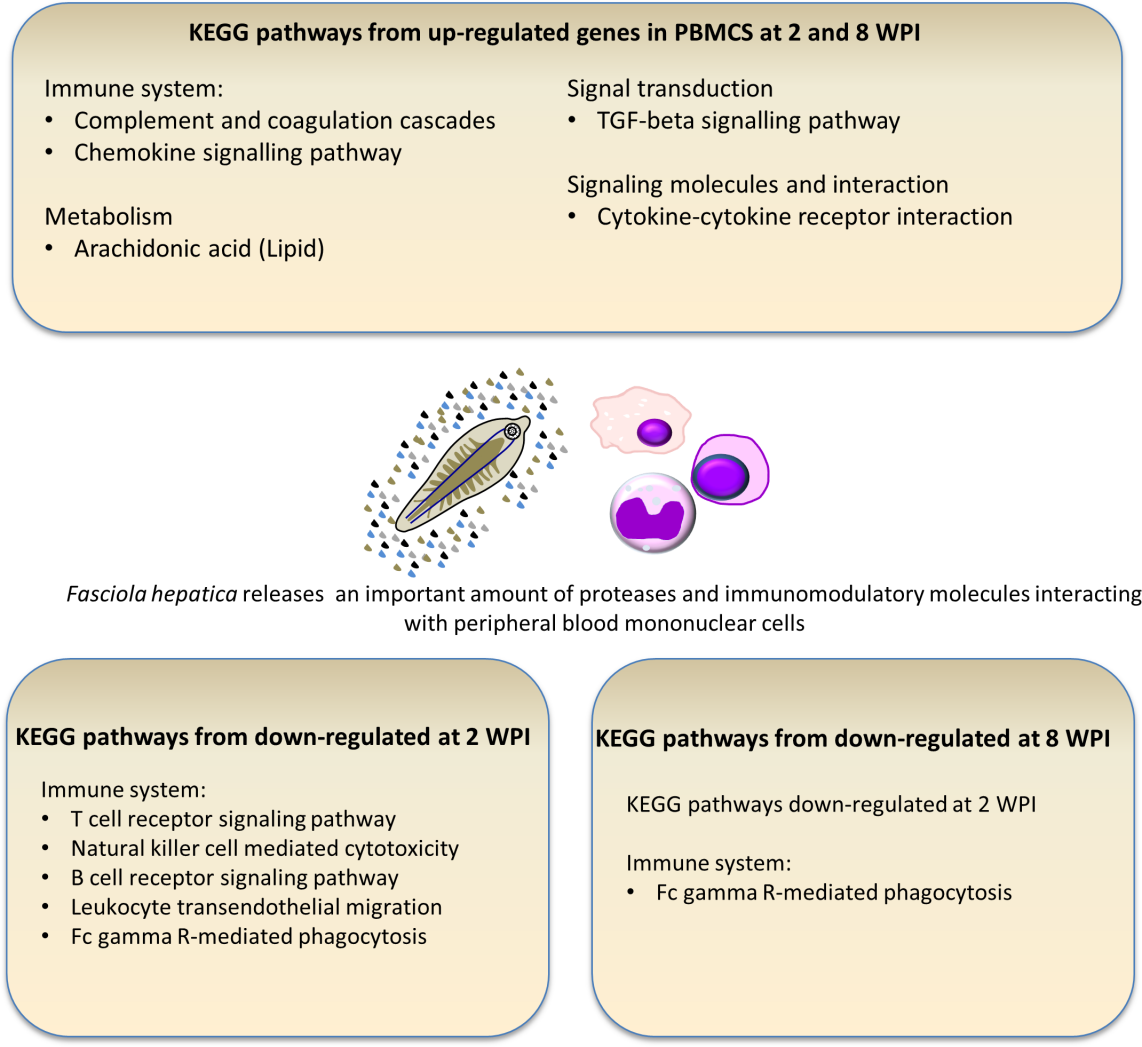
Schematic representation showing KEGG enriched pathways in up-and down-regulated genes from ovine PBMCs after 2 and 8 weeks post infection with *Fasciola hepatica*.

We explored the functional roles of the DTGs at 2 and 8 WPI. **Table 2** shows overrepresented GO terms (biological processes) for up- and down-regulated genes at 2 and 8 WPI. Similarly, **Table 3** shows the overrepresented GO terms (molecular function) for up- and down-regulated genes at 2 WPI. There was no overrepresentation of GO terms related to molecular function at 8 WPI. **Fig 2** shows a schematic representation of significantly (*p*<0.05) enriched KEGG pathways in up-and down-regulated genes from ovine PBMCs at 2 and 8 WPI.

**Table 2:** Biological processes terms (Gene ontology) overrepresented for UP- and DOWN-regulated genes in ovine peripheral blood mononuclear cells (PBMCs) after 2 and 8 weeks post infection with *Fasciola hepatica*.

**At 2 WPI**				
**GO biological process**	**Fold Enrichment**	**P value**	**UP**	**DOWN**
lipoxygenase pathway	42.7	2.38E-02	GPX4, LTC4S, ALOX5AP, ALOX5	
positive regulation of alpha-beta T cell proliferation	36.52	2.79E-03	LGALS9, HLA-A, EBI3	ZAP70, CD28
positive regulation of T cell activation	6.67	2.71E-02	LGALS9, HLA-A, AIF1, HLA-DRB1, CCL2, TGFB1, EBI3, PYCARD	ZAP70, CD28
regulation of interferon-gamma production	10.44	4.97E-02	LGALS9, HLA-A, HLA-DRB1, EBI3, PYCARD, PGLYRP1	CCR7
humoral immune response	8.58	7.44E-04	HLA-A, C1QB, FCN2, BST2, HLA-DRB1, CCL2, C4BPA, EBI3, C1QC, ANG	CD28
response to lipopolysaccharide	6.23	6.38E-04	PF4V1, LGALS9, MGST1, HCK, CD300LB, LTC4S,S100A8, CCL2, TGFB1, PYCARD, NR1H3, CCR7, CEBPE	CD27
leukocyte migration	5.8	3.24E-02	PF4V1, S100A9, S100A12, HCK, CCL23, S100A8, CCL2	PLCG1, L1CAM, ZAP70, CCR7
positive regulation of cytokine production	4.71	4.04E-02	CLEC6A, PRG3, LGALS9, HLA-A, NFAM1, CCL2,TGFB1, EBI3, PYCARD, CLEC4E, SPTBN1	CD28, CCR7
innate immune response	4.01	2.86E-07	PLCG1, CLEC6A, LGALS9, HLA-A, C1QB, FCN2, S100A9, S100A12, TYROBP, BST2, HCK, CD300LB, CCL23, AIF1, S100A8, DUSP6, HLA-DRB1, CCL2, TGFB1, C4BPA, PADI4, PYCARD, CLEC4E, C1QC, PGLYRP1	RNF125, SPTBN1, ZAP70, CD28, DUSP7
**At 8 WPI**				
**GO biological process**	**Fold Enrichment**	**P value**	**UP**	**DOWN**
lipoxin metabolic process	> 100	1.18E-02	ALOX5AP, ALOX5, ALOX15	
leukotriene metabolic process	49.37	5.78E-04	PRG3, ALOX5AP, ALOX5, ALOX15	CYP4F3
negative regulation of immune response	15.74	2.05E-02	LGALS9, LILRB2, C4BPA, ALOX15, DUSP10	IRG1

**Table 3:** Molecular function terms (Gene ontology) overrepresented for UP- and DOWN-regulated genes from ovine peripheral blood mononuclear cells (PBMCs) after 2 weeks post infection with *Fasciola hepatica*.

GO molecular function	Fold Enrichment	P value	UP	DOWN
arachidonic acid binding	69.38	3.40E-02	S100A9, S100A8, ALOX5AP	
RAGE receptor binding	50.46	3.95E-03	S100A9, S100A12, S100A8, S100A4	
cytokine receptor binding	5.53	1.63E-02	PF4V1, SMAD2, LRG1, CCL23, CCL2, TGFB1, EBI3, PYCARD, TFF2, ECM1	PLCG1

### Gene transcription in PBMCs at 2 WPI

At 2 WPI, the juvenile flukes are between 1 to 2.5 mm in length, and they can be found penetrating the liver capsule and burrowing into the parenchyma, causing sub-capsular haemorrhage with eosinophil and macrophage infiltration [2, 32, 33].

Overall, from the total number of DTGs at 2 WPI, 115 genes were enriched in transcription at relative to other time-points and/or uninfected controls. We found no evidence of specific biological pathways up-regulated at 2 WPI that were not also up-regulated at 8 WPI; however, we did see a number of DTGs that were specifically up-regulated at this earlier stage of infection. “T-cell activation” was the most notable GO term (biological process) over-represented for DTGs at 2 WPI. A number of proteins have been identified to play a role in the T-cell activation in the literature [34]. Among these are the galectins, which are part of a family of animal lectins that bind N-acetyllactosamine-containing glycans. Galectins also have roles in pathogen recognition, shaping the course of adaptive immune responses and fine-tuning the inflammatory response [35]. In our study, two isoforms of galectin-9 (transcripts ENSOART00000017447 and ENSOART00000001365) were found to be up-regulated at 2 and 8 WPI. There is evidence galectins are part of the host-parasite interaction, for example promoting adhesion and changing mucus properties during parasite infection [20] or directly interacting with the parasite, as is the case of galectin-14 and galectin-11 with *F*. *hepatica* in the bile ducts [20]. We have previously demonstrated the up-regulation of the same genes in ovine liver parenchyma infected with *F*. *hepatica* [21]. An important role for galectins is their involvement in T-cell differentiation: galectin-9 together with TGF-beta1 have synergistic effects on the rate of conversion of inflammatory to regulatory T-cells *in vitro* inducing phosphorylation of SMAD2/3, ERK1/2 and formation of the SMAD2/3-SMAD4 complex [36]. The activation of SMAD3 has also been involved in the expression of Galectin-9 together with TGF-β [37]. Interestingly, we found *Smad2* and *tgf-β1* were up-regulated at 2 WPI. *Smad2* and *Smad3* are essential for the TGF-beta-mediated regulation of T-cell plasticity [38]. Elevated levels of peripheral blood mononuclear cell (PBMC)-derived transforming growth factor (TGF)-β1 have been observed in early phases of the infection with *F*. *hepatica* in cattle [17]. *F*. *hepatica*-induced TGF-β plays a critical role in bystander suppression of autoantigen-specific Th1 and Th17 responses that mediate autoimmune diseases [39].

Aside from galectins, other lectins also play an important role in the immune response [40, 41]. GO analysis revealed an over-representation of “positive regulation of cytokine production” and “innate immune response” involving (amongst others) three genes encoding C-type lectin receptors (CLRs; *cleC6A*, *cleC4E* and *cleC3B*). These receptors are crucial for tailoring immune responses to different pathogens, triggering distinct signalling pathways that induce the expression of specific cytokines which determine T-cell polarization [42, 43]. CLEC4E (also known as MINCLE) induces inflammatory cytokine production and drives the infiltration of neutrophils into damaged tissue [44]. This is very interesting since the migration of *F*. *hepatica* in liver produces extensive damage and subsequent cellular infiltration; our date suggests that CLEC4E may be an important driver of in the extensive liver damage and subsequent cellular infiltration associated with the juvenile migration of *F*. *hepatica*. Considering the comparatively moderate damage caused by *Fasciola* juveniles in cattle during the acute phase [2, 3], it would also be interesting to explore the transcriptional profile of the *cleC4E* (*mincle*) ortholog in cattle during the migration of juvenile flukes through liver tissue. A role for different C-type lectins receptors from macrophages (mannose receptor and Dectin-1,) has been described in response to *F*. *hepatica* antigens [45]. CLRs clearly merit further study with respect to the progression of the response to *F*. *hepatica* infection in sheep and other natural hosts.

Another important group of proteins with immune function that was shown to be up-regulated at 2 WPI are the S100 proteins, which are involved in “leukocyte migration” and the “innate immune response” (Table 2). The S100 protein family consists of 24 members comprising a group of proinflammatory molecules released by phagocytes [46, 47]. Six members of this family were up-regulated in PBMCs at 2 WPI (S100A4, A8, A9, A10, A11 and A12). S100A8, A9 and A12 S100A12 belong to the calgranulin subfamily and are known to be involved in host-responses to, for example, *Echinococcus granulosus* [48], *Plasmodium falciparum*[49], *Leishmania major* [50], *Eimeria brunetti* and *E*. *acervulina* [51] and *F*. *hepatica* [21]. Our analysis of GO terms for molecular function shows significant overrepresentation of “RAGE receptor binding” (**Table 3**). RAGE (i.e. receptor for advanced glycation end products) is a multi-ligand receptor of the immunoglobulin family that plays a role in the activation and differentiation of T-cells [52, 53] and has been shown to transduce the extracellular effects of S100A4, S100A11, S100A12 and other S100A proteins [54]. Further studies are necessary to elucidate the role of S100 proteins and RAGE in T-cell differentiation and other aspects of the host immune response to parasitic infection.

Also interesting among genes up-regulated specifically at 2 WPI is *gata-1*. GATA-1 expression suppresses the development of the Th1 response and transcription of associated genes (e.g., *ifnɣ* and *cxcr3*) in human T-cells [55]. Other up-regulated genes at 2 WPI, include four isoforms of *cd300*. CD300s are type I transmembrane glycoproteins with a single IgV-like extracellular domain [56]. They are crucial to remodelling host tissue and contribute to tuning, directing or terminating active immune responses. This is highly relevant in a disease, like fascioliasis, that involves the remodelling of the liver tissue, especially in the initial stages of the infection.

By comparison, *Fasciola* infection related to relatively few down-regulated genes generally. Among down-regulated genes at 2 WPI, we identified enrichment of genes associated with “T cell receptor signaling pathway”, “NTK cell cytotoxicity”, “B-cell receptor” and “Leukocyte transendothelial migration” pathways. For the T-cell receptor and NTK cytotoxicity signaling pathway the gene encoding the ZP70 was down-regulated, this is an essential kinase in initiating T-cell responses by the antigen receptor [57]. For the “B cell receptor pathway” the gene encoding CD22 was found down-regulated. CD22 is a member of the Siglec (sialic-acid-binding immunoglobulin-like lectin) family, this is an accessory co-receptor expressed on the B-cell surface that negatively modulates the B cell antigen receptors [58]. This suggests that this down-regulation of CD22 might result in a positive regulation of B cell antigens receptors in *F*. *hepatica* infection at 2 WPI. The infection with *F*. *hepatica* brings as a consequence an increase in transendothelial migration towards the damaged tissue in liver after migration of the liver fluke. Therefore a down-regulation in this mechanism should be elicited by the immune system [59]. It has been described that fatty acid from *F*. *hepatica* (Fh12) binds protein inhibiting TLR4 activation and suppresses the inflammatory cytokines [60]. Other components of the *F*. *hepatica* tegument also have anti-inflammatory functions in vitro suppressing dendritic cell maturation and function [61, 62].

### Gene transcription in PBMCs at 8 WPI

At 8 WPI, migrating juvenile flukes should be about 10 mm in length and most will be entering the bile ducts, or migrating through the left liver lobe [2]. In the liver it is possible to find haemorrhagic areas, hepatic hyperplasia and fibrosis as part of the tissue repair process. Infiltrating lymphocytes and eosinophils will still be present at 8 WPI, suggesting that the parasites will still have close contact with immune cells, however this is more restricted compared with the situation at 2 WPI. By 8 WPI, relative to early-stages of infection, we found enrichment among up-regulated DTGs for biological processes including: “lipoxin metabolic process”, “leukotriene metabolic process” and “negative regulation of immune response” (**Table 2**). In the first two processes, the proteins ALOX5, ALOX5AP and ALOX15 play an important role in the production of lipoxins, which are members of a family of bioactive products generated from arachidonic acid [63]. Lipoxins have been described as playing a role in "immunomodulation" in *Toxoplasma gondii* infection [64] and suggested to influence T-lymphocyte effector functions in the setting of polarized T-helper cell responses (Th1 and Th2) [63]. In a previous study [21] we identified 572 gens up-regulated in liver tissue 8 WPI post infection with F. hepatica, forteen of these genes were identified to be up-regulated in PBMCs at this time point. None of the forty two down-regulated genes identified in liver at 8 WPI [21] were identified to be down-regulated in PBMCs at this time point in this study.

### Gene transcription in PBMC is constitutively altered with *Fasciola* infection

In addition to DTGs found at 2 or at 8 WPI, we described a large number of genes differentially transcribed throughout (i.e. constitutively) *F*. *hepatica* infection in sheep. An overall appraisal of these DTGs showed KEGG and GO enrichment related to the immune system (**Table 2**), including “positive regulation of T-cell activation”, “regulation of interferon-gamma production”, “Leukocyte migration”, “positive regulation of cytokine production” and the “innate immune response”. Of particular interest are the up-regulation, at 2 and 8 WPI, of the same genes involved in processes including: “complement and coagulation cascades”, “chemokine” and the “TGF-beta signaling pathways” (**Fig 2**).

Three genes encoding proteins related with the complement system were up-regulated at 2 WPI: complement component 1, q subcomponent, B chain (*c1qb*) and q subcomponent, C chain (*c1qc*) and complement component 4 binding protein, alpha (*c4bpa*). These representatives of the complement system exert a variety of functions over immune cells. For example, complement activation fragments such as C4b interact with complement receptors and regulators modulating T-cell lineage and cytokine production [65]. C4BP also induces B-cell proliferation [66]. Complement inhibition is crucial for parasite survival within the host tissue or, were relevant, during blood-feeding [67]. Previous studies suggest that extracts from *F*. *hepatica* adults inhibit both the classical and alternative pathways of complement activation in normal bovine and human sera [68]. Similarly, a reduction in the classical pathway of complement-mediated lysis by normal rat sera has been described using extracts from metacercariae, eggs and E/S products [69]. Our results suggest that the parasite has an influence over the complement activation pathways and this deserves future investigations.

It is well known that the *F*. *hepatica* infection will attract numerous inflammatory cells to the liver [2]. Among the genes involved in these processes, *ccl2* and *ccl23* were differentially transcribed at both 2 and 8 WPI. CCL2, also known as monocyte chemoattractant protein-1 (MCP-1), is a member of the C-C chemokine family and a highly potent chemo-attractor of monocytes and CD41 T-cells. Monocytes/macrophages are found to be the major source of CCL2 in the peripheral blood [70]. Unlike the other C-C chemokines, CCL2 regulates Th-cell differentiation by polarizing Th0 cells toward a Th2 phenotype [71]. CCL2 is released locally in response to gastrointestinal nematode infection in mice and has been implicated in resistance to *Trichuris muris* by steering the host towards a Th2-type response [72]. CCL23 has been related to suppression of the production and release of neutrophils from the bone marrow [73]. Considering the important role that neutrophils play in the host response to many metazoan parasites [74–76], including *F*. *hepatica* [77, 78], it maybe that up-regulation of CCL23 is a consequence of an immunomodulatory influence by *F*. *hepatica* to block neutrophil production; this is the first suggestion of this kind of interaction with this parasite in sheep.

Constitutive changes in the metabolism of PBMCs in relation to “arachidonic acid” are also notable. At 2 WPI, we found up-regulation of arachidonate 5-lipoxygenase (*alox5*), arachidonate 5-lipoxygenase-activating protein (*alox5ap*) and *alox15* in PBMCs. Interestingly, *alox5ap* and *alox5* were also found to be up-regulated in liver tissue at 8 weeks after infection [21]. ALOX5 oxidizes arachidonic acid to produce 5-hydroperoxy-6,8,11,14-eicosatetraenoic acid, which is an intermediate for various leukotrienes of lipid mediators [79]. It has a pivotal role in the regulation of specific antibody responses by managing primary B-cells and Tfh (follicular) cells *in vivo* [80]. It has also suggested that 5-Lipoxygenase negatively regulates Th1 response during *Brucella abortus* infection in mice [81]; an up-regulation of 5-lipoxygenase-activating protein here appears to point to another pathway supporting Th1 suppression in *F*. *hepatica* infected sheep.

“Fc gamma R-mediated phagocytosis” was the only biological pathway reduced at both 2 and 8 WPI. This pathway is associated with phagocytosis of antibody-coated pathogens. Phagocytosis is commonly reduced with alternative activation of macrophages [82], which is a common hallmark of helminth-induced immunomodulation [83] and mediated by *Fasciola hepatica*, specifically, at least in part through the parasite secretion of thioredoxin peroxidase [84].

Surprisingly, a high number of genes encoding interleukins did not show any transcriptional activity at any of the time points examined in infected or control animals. This suggests that transcription of interleukins is reduced or non-existent in peripheral blood in animals and/or interleukins are only transcribed in cells that are directly interacting with the parasite. Interestingly, it has been recently described that tegumental antigens from *F*. *hepatica* induce unresponsive or anergic-like T cells in a mouse model [85], which might be related with our findings. In the case of interleukins considered to be typical of a Th2 response (IL4, IL5 and IL13) transcription levels were reported only at some time points and insufficiently to detect statistically-significant differences in transcription here. IL10 transcription levels were reported at all the time points studied, however no significant differences in transcription were found.

## Conclusions

To our knowledge this is the first RNA-seq study to examine gene transcription in PBMCs from a natural host (sheep) infected with *F*. *hepatica*. We previously reported the exploration of the transcriptome of ovine liver tissue infected with the liver fluke [21]. The current study focused on the temporal progression of the host response to *Fasciola*, examining changes in the transcriptional profile of peripheral blood mononuclear cells (PBMCs) through the first 8 weeks of infection in the same animals used in our previous study. The results presented identify key genes involved in the immune processes activated by the presence of the parasite in PBMCs. Important biological events seem to be modified for the presence of the parasite, including the complement system, the chemokine signaling, T-cell activation and metabolic processes that influence the immune response. Important groups of genes appear to interact with, or at least be stimulated in response to the parasite including lectins, S100 and CD300. We also identify a number of genes that are differentially transcribed at the 2 time points examined suggesting that they can be used as markers of infection in PBMCs. Most changes in PBMC are largely uniform across the 8 weeks of infection. Some changes, e.g., positive regulation of T-cell activation and leukocyte migration, are specific to early infection. Others, relating more to lipoxin metabolic process and Fc gamma R-mediated phagocytosis appear directed towards later stage infection. The next steps in the investigation of host/parasite interaction between sheep and the liver fluke should involve the development of tools to measure the expression of proteins from differentially transcribed genes. These tools should contribute to a better knowledge of the interaction between host and parasite and also to a better understanding of the kind of immune response elicited by the parasite.

## Supporting Information

S1 Fig Venn diagrams showing consensus of found using both strategy 1 and 2 from material and methods. (TIF)

S1 Table Number of RNA-seq reads from control and infected animals. (XLSX)

S2 Table **A** shows the differentially transcribed genes in each infected animal using NOISeq-sim comparing transcription levels at pre infection (T1) with 2 weeks post infection (T2). Also it shows the number of DTGs that were found in at least three animals and number of DTGs that were found uniquely in infected and not in control animals. **B** shows the results for the comparison between pre infection (T0) and 8 weeks post infection (T8). **C** shows the number of DTGs after comparing control and infected animals at transcription level at 2 weeks post infection (T1) with 8 weeks post infection (T2)
(XLSX)

S3 Table List of up-regulated genes and TPM (transcripts per million) values in PBMCs from sheep at 2 weeks post infection with *Fasciola hepatica*. (XLSX)

S4 Table List of DOWN-regulated genes and TPM (transcripts per million) values in PBMCs from sheep at 2 weeks post infection with *Fasciola hepatica*. (XLSX)

S5 Table List of up-regulated genes and TPM (transcripts per million) values in PBMCs from sheep at 8 weeks post infection with *Fasciola hepatica*. (XLSX)

S6 Table List of DOWN-regulated genes and TPM (transcripts per millions) values in PBMCs from sheep at 8 weeks post infection with *Fasciola hepatica*. (XLSX)
