# Supplementary figures and images for: Time-Course Study of the Transcriptome of Peripheral Blood Mononuclear Cells (PBMCs) from Sheep Infected with Fasciola hepatica
Source: PLoS One. 2016 Jul 20;11(7):e0159194. doi: 10.1371/journal.pone.0159194 (PMC4954650; doi:10.1371/journal.pone.0159194)

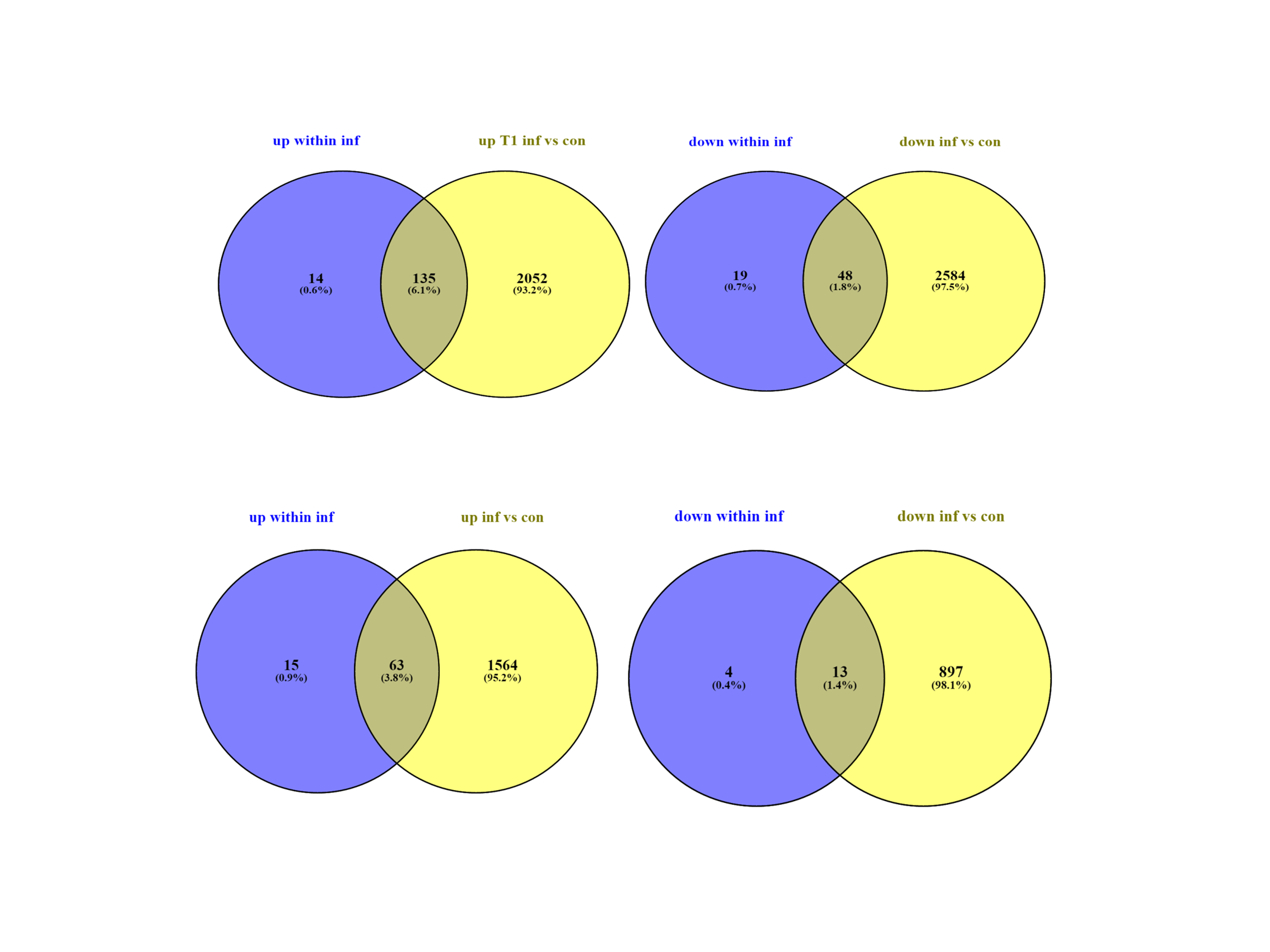

Supplement: S1 Fig — (TIF) [file pone.0159194.s001.tif]
